# A systematic review and meta-analysis of neurological soft signs in relatives of people with schizophrenia

**DOI:** 10.1186/1471-244X-11-139

**Published:** 2011-08-22

**Authors:** Kishen Neelam, Deepak Garg, Max Marshall

**Affiliations:** 1 Lantern centre, University of Manchester, Vicarage Lane, Preston, PR2 8DY, UK; 2 Greater Manchester West Mental Health NHS Foundation Trust, Bury New Road, Prestwich, Manchester, M25 3BL, UK; 3 Lancashire Care NHS Foundation Trust, Walton Summit, Preston, PR5 6AW, UK; 4 Humber NHS Foundation Trust, Clarendon Health Centre (Victoria House), Park Street, Hull, HU2 8TD, UK

## Abstract

**Background:**

Neurological soft signs are subtle but observable impairments in motor and sensory functions that are not localized to a specific area of the brain. Neurological soft signs are common in schizophrenia. It has been established that soft signs meet two of five criteria for an endophenotype, namely: association with the illness, and state independence. This review investigated whether soft signs met a further criterion for an endophenotype, namely familial association. It was hypothesized that if familial association were present then neurological soft signs would be: (a) more common in first-degree relatives of people with schizophrenia than in controls; and (b) more common in people with schizophrenia than in their first-degree relatives.

**Method:**

A systematic search identified potentially eligible studies in the EMBASE (1980-2011), OVID - MEDLINE (1950-2011) and PsycINFO (1806-2011) databases. Studies were included if they carried out a three-way comparison of levels of soft signs between people with schizophrenia, their first-degree relatives, and normal controls. Data were extracted independently by two reviewers and cross-checked by double entry.

**Results:**

After screening 8678 abstracts, seven studies with 1553 participants were identified. Neurological soft signs were significantly more common in first-degree relatives of people with schizophrenia than in controls (pooled standardised mean difference (SMD) 1.24, 95% confidence interval (c.i) 0.59-1.89). Neurological soft signs were also significantly more common in people with schizophrenia than in their first-degree relatives (SMD 0.92, 95% c.i 0.64-1.20). Sensitivity analyses examining the effects of age and group blinding did not significantly alter the main findings.

**Conclusions:**

Both hypotheses were confirmed, suggesting that the distribution of neurological soft signs in people with schizophrenia and their first-degree relatives is consistent with the endophenotype criterion of familial association.

## Background

Neurological soft signs are subtle but observable impairments in motor and sensory functions that are not localized to a specific area of the brain nor characteristic of any specific neurological condition [1]. Typically they are classified into signs relating to: motor co-ordination, sequencing of complex motor tasks, sensori-motor integration, and disinhibition [2]. Neurological soft signs are known to correlate with a range of neuro-cognitive and neuro-anatomical abnormalities, and it has been proposed that they represent an underlying defect in neural integration [3]. Until recently neurological soft signs have been considered of little practical clinical significance, but the prospect that they could be an endophenotype of schizophrenia has led to a resurgence of interest [4].

Endophenotypes are defined as trait-markers that are present independent of the manifestation of a disease [5]. They represent a phenotype "within" the patient below the level of overt behavioural or psychopathological symptoms. Five criteria for an endophenotype have been put forward: (i) association with illness (higher rates of endophenotype in people with the illness than that found in the general population); (ii) state independence (presence of endophenotype irrespective of the disease state); (iii) familial association (the endophenotype is present at higher rates in unaffected family members than in the general population); (iv) co-segregation (higher prevalence of the endophenotype in ill relatives of ill probands than in well relatives of ill probands); and (v) heritability (the extent of variation of the endophenotype that is attributable to the genetic variation) [6]. It has been proposed that endophenotypes may be of particular value to genetic research on mental disorders because they are more closely related to underlying gene expression than is psychopathology [7]. For example, endophenotypes could be used for the discovery of genes associated with schizophrenia [8].

Neurological soft signs are a potential endophenotype for schizophrenia, because: they are common in people with the disorder (ranging from 50 to 65% of people with schizophrenia [1], predate the onset of the disorder [9], and can be plausibly linked to the underlying brain abnormalities postulated by the neurodevelopmental theory of schizophrenia [1,10]. It has been established, in a systematic review and meta-analysis [3], that neurological soft signs in schizophrenia meet two criteria for an endophenotype: association with illness (because they occur much more frequently in people with schizophrenia than in controls); and state-independence (because they are present whether or not the illness is active).

This systematic review and meta-analysis aimed to determine how far soft signs in schizophrenia met a further criterion for an endophenotype, namely familial association. It was hypothesized that if familial association were present then neurological soft signs would be: (a) more common in first-degree relatives of people with schizophrenia than in controls; and (b) more common in people with schizophrenia than in their first-degree relatives.

## Methods

### Data Sources

The search strategy aimed to identify all studies that had conducted three-way comparisons of neurological soft signs between people with schizophrenia, their first-degree relatives, and normal controls. People with schizophrenia were defined as having been given a diagnosis of schizophrenia, or schizophrenia-like disorder on the basis of a standardised diagnostic assessment. Three-way comparisons were considered more suitable for testing our hypotheses than two way comparisons because differences in effect sizes between comparisons (e.g. relatives versus controls and schizophrenia versus relatives) would not be confounded by the use of different assessment techniques, raters, or instruments.

Unlike randomised controlled trials, such comparative studies are not well indexed; therefore a search strategy was generated empirically by examining the indexing of relevant papers from the authors' personal databases and from the references of previously published reviews [1,11,12]. This search strategy was designed to be sensitive rather than specific, and was applied to the following databases: EMBASE, OVID-MEDLINE and PsycINFO. The sensitivity of the search was confirmed by checking the reference lists of the identified studies and reviews to ensure that no relevant papers had been omitted. Where an omission had occurred, the indexing of the omitted paper was scrutinized, and the search strategy was modified and re-run. This process continued until no new papers were identified. The original search performed in September 2009 was updated again in April 2011 (see table 1).

**Table 1:** Search strategy

1	neurological$.ab, kw, rt, tm, ti.	278114
2	(neuro$ adj3 sign#).ab, kw, rt, tm, ti.	22797

3	soft sign#.ab, kw, rt, tm, ti.	1109

4	(soft adj2 neuro$).ab, kw, rt, tm, ti.	1411

5	NSS.ab, kw, rt, tm, ti.	2494

6	SNS.ab, kw, rt, tm, ti.	4661

7	1 or 2 or 3 or 4 or 5 or 6	294985

8	exp schizophrenia/	244555

9	(schizo$ or psychotic$ or psychosis or psychoses or hebephreni$ or oligophreni$).ab, kw, rt, ti.	365517

10	((CHRONIC$ or SEVER$) adj5 MENTAL$ adj5 (ILL$ or DISORDER$)).ab, kw, rt, ti.	18088

11	first episode.ab, kw, rt, ti.	15961

12	Prodrom$.ab, kw, rt, ti.	9987

13	8 or 9 or 10 or 11 or 12	434722

14	7 and 13	8678

### Study Selection

KN screened each abstract, and copies of any potentially relevant articles were obtained. KN and DG independently reviewed the articles and any disagreements in selecting the studies between them were resolved by discussion. Unresolved disagreements between KN and DG were resolved by discussion with the third reviewer (MM). Studies were included if they compared levels of soft signs between normal controls, first-degree relatives of people with schizophrenia and people with schizophrenia within the same study design.

### Data Extraction

The outcome variable for the review was the mean number of neurological soft signs. Data were excluded if: they only referred to subsets of the soft signs family (such as frontal release signs), they were combined with numbers of hard signs, or were reported exclusively in a categorical format (as there is no universally agreed cut off point for presence or absence of soft signs) [13].

Several scales are available for rating the number of soft signs, the most well known being: the Neurological Evaluation Scale [2], the Condensed Neurological Examination or Rossi Scale [14], and the Standardised Neurological Examination [15]. Since there is considerable overlap between these scales, data were included if any of these three measures were used [13].

Demographic and study variables were extracted and reported in tabular form, including: age, sex, number of years in education, illness duration, type of control group, and type of relative (primary or secondary degree, or if primary degree: sibling, offspring, parent or mixed group). In addition, each included study was rated on three quality criteria: evidence of inter-rater reliability on the ratings of soft signs; rater blind to the status of the participant (although adequate blinding is difficult to attain and this bias cannot be fully eliminated); and degree of age matching between comparison groups (see table 2). Data were extracted independently by two reviewers (KN and DG) and crosschecked by the double entry method. Disagreements were resolved by discussion and involvement of the third reviewer (MM).

**Table 2:** Description of included studies

Study reference	NSS Scale	Blindness#	Schizophrenia Group							Relatives Group						Control Group					
			**Disorder (Diagnostic method)**	**N**	**Mean Age**	**Male%**	**Years in education**	**Illness duration in years**	**NSS Mean (SD)**	**Relative Type**	**N**	**Mean Age**	**Male%**	**Years in education**	**NSS Mean (SD)**	**Control Type**	**N**	**Mean Age**	**Male%**	**Years in education**	**NSS Mean (SD)**

Compton et al 2007 [24]	NES	No	Scz or scz-like (SCID-DSMIV)	73	32.4	58%	13.1	nr	20.7 (10.6)	Mixed	44	43.2	16%	13.1	15.9 (9)	Waiting area and public	54	44.8	50%	12.3	14.3 (8.3)

Egan et al 2001 [25]	NES	Yes	Scz (DSMIV	115	35.9	83%	15.4	nr	6.8 (4.24)	Siblings	185	36.3	43%	15.4	3.05 (2.82)	Normal Volunteers	88	33.4	42%	15.9	2.8 (2.29)

Gourion et al 2003 [26]	SNE	No	Scz or scz-like (DSMIV)	18	28.2	67%	5.5	9.1	18.7 (9.4)	Parents	36	60.4	50%	5.5	16 (5.8)	Hospital Staff or volunteers	42	26.6	38%	7.1	3.9 (2.8)

Ismail et al 1998 [27]	CNE	No	Scz (DSMIIITR)	60	38.2	73%	nr	14.8	3.25 (3.31)	Siblings	21	37.9	nr	nr	1.33 (2.01)	Normal workers	75	35.9	79%	nr	0.2 (0.54)

Mechri et al 2009 [28]a	SNE	No	Scz (DIGS)	69	28.2	74%	12.4	6.1	15 (7.9)	Siblings	43	29.2	40%	13.8	8 (4)	Hospital Volunteer	108	28.2	63%	13.5	5.8 (3.3)

Mechri et al 2009 [28]b	SNE	No	Scz (DSMIV)	66	31.2	76%	8.5	nr	19.5 (5.2)	Siblings	31	32.2	71%	10.6	10.8 (3.4)	Hospital Volunteer	60	30.8	67%	9.8	4.2 (2.1)

Rossi et al 1990 [14]	CNE	Yes	Scz (DSMIII)	58	34.8	64%	6.9	11.1	12.63 (4.79)	Mixed	31	39.2	58%	6.9	9.8 (2.42)	Family Practice	38	36.2	58%	8.5	4.07 (2.53)

Yazici et al, 2002 [29]	NES	No	Scz (DSMIV)	99	31.4	48%	10.9	8.9	20.47 (10.07)	Siblings	80	31.6	53%	10.9	10.6 (7.23)	Normal Volunteers	59	31.6	53%	10.8	6.66 (5.37)

NSS - Neurological soft signs, NES - Neurological Evaluation Scale, SNE - Standardised Neurological Examination, CNE - Condensed Neurological Examination, Scz - Schizophrenia, Scz-like - Schizophrenia-like disorders, DSM- Diagnostic and Statistical Manual, SCID- Structured Clinical Interview for DSM, SCAN- Structured Clinical Assessment in Neuropsychiatry, DIGS - Diagnostic Interviews for Genetic Studies, nr - not reported, # - All studies reported inter-rater reliability.

### Data synthesis

The data were analysed using Comprehensive Meta-Analysis version 2, a dedicated meta-analysis programme (BioStat, Inc, Englewood, NJ). The analysis was based on all included studies and consisted of three comparisons: first-degree relatives of people with schizophrenia versus normal controls; people with schizophrenia versus normal controls; and people with schizophrenia versus first-degree relatives.

The standardized mean difference was calculated for each comparison. The standardized mean difference (SMD) is a clinically useful effect size defined as the difference in means between two groups standardized by dividing by the with-in groups' pooled standard deviation. The SMD effect size can be interpreted as the average percentile standing of the mean in the comparison group relative to the mean in the control group. Thus, an effect size of "0" indicates that the mean in the comparison group is at the 50^th ^percentile of the control group, and the distribution of scores in the comparison group completely overlaps with the distribution of scores in the control group. An effect size of 0.8 indicates that the mean in the comparison group is at the 79^th ^percentile of the control group, and shows a non-overlap of nearly 50% in the distribution of scores between the two groups. Cohen has defined a standardised mean difference of 0.2 as small, 0.5 as medium, and 0.8 as large [16].

The results of the comparisons were illustrated in a Forest plot, in which the standardised mean difference for each study and the associated 95% confidence intervals were plotted on a horizontal axis ranging from -1 to 4. All comparisons were tested for heterogeneity using the I^2 ^statistic. When significant heterogeneity was present, the cumulative standardised mean difference was calculated using random effects. When significant heterogeneity was present, and sufficient studies were available (greater than 9), meta-regression was used to determine whether the heterogeneity could be explained by moderator variables, such as: age, number of years in education, use of anti-psychotic medication or illness duration.

Scores on measures of neurological soft signs are thought to increase with age [14,17], and may be sensitive to inadequate rater training, or rater bias. Therefore sensitivity analyses were conducted for each of the three comparisons that excluded studies that: had more than a decade age difference between comparison groups; failed to provide evidence of inter-rater reliability; or used raters that were not blind to the group allocation of participants. The possibility of publication bias was examined using the Orwin fail-safe N.

## Results

**Figure 1:**
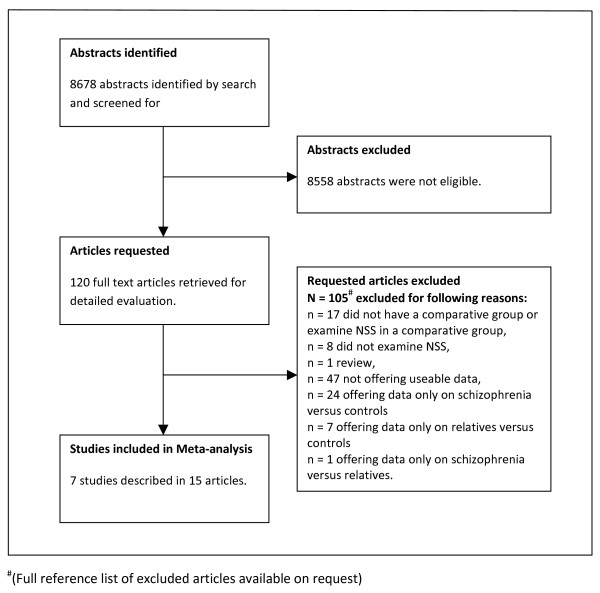
**Flowchart of meta-analysis**.

The search strategy identified 8678 articles, of which 120 referred to studies that were thought to potentially satisfy the inclusion criteria. After obtaining the full text of these articles, it was found that 105 referred to studies that did not meet inclusion criteria. Seven studies, described in 15 articles, met inclusion criteria and offered data for the meta-analysis [14,18-31] (for details see study flowchart Figure 1).

All seven included studies compared levels of soft signs amongst people with schizophrenia, relatives, and normal controls, within the same design, using the same assessment method and the same raters. The participants in the included studies comprised: 558 people with schizophrenia or schizophrenia-like disorders, 471 first-degree relatives (there were no studies of second degree relatives), and 524 normal controls (see table 2). One study [28] included two independent samples from different countries (France and Tunisia). Within the meta-analysis, this study was treated as two separate studies. Of the remaining six studies the country of origin of two studies was USA [24,25] and there was one study each from Sweden [27], Italy [14], Turkey [29] and France [26].

The Neurological Evaluation Scale [2] was used in 3 studies, whilst the Rossi Scale [14] and the Standardised Neurological Examination [15] were each used in 2 studies (see table 2).

The group of first -degree relatives was categorised as a "mixed" group in 2 studies [14,24] as they included a combination of offspring, siblings and/or parents. In the first of these studies [14], the ages of all three groups were similar, suggesting that the relatives group were principally siblings and that there was little chance of an age difference between groups confounding the comparison. In the second study [24], the relatives group were 11 years older on average than the schizophrenia group, whilst the control group were 12 years older on average. This suggested a risk of an age confound, as neurological soft signs could increase with age. In 4 studies the first degree relatives group consisted only of siblings [25,27-29] and in one study consisted only of parents [26]. In this study, parents were on average 32 years older than people with schizophrenia, whereas controls were 10 years older, again suggesting the possibility of an age confound.

### Neurological soft signs in first-degree relatives versus controls

**Figure 2:**
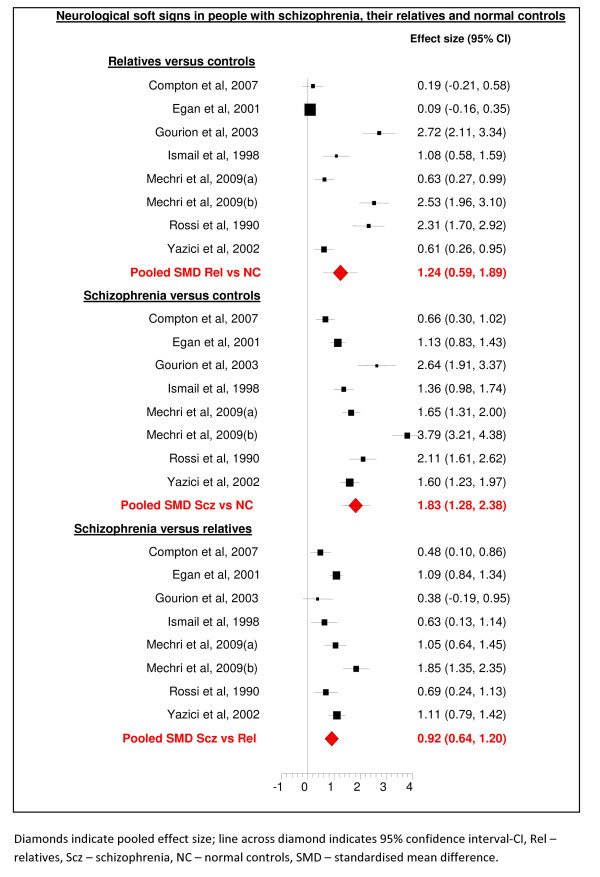
**Forest plot of neurological soft signs**.

This comparison comprised 995 participants from seven studies (see Figure 2). Whilst all seven studies showed the same direction of effect, heterogeneity between studies was significant (I^2 ^94.9, p < 0.001), so the data were analysed using a random effects model. As the comparison included less than 9 studies, meta-regression was not attempted. The pooled SMD (random effects model) was 1.24 (95% confidence interval 0.59 to 1.89). This indicated a large and significant effect size. This finding was stable (SMD 1.03 95% confidence interval 0.42 to 1.63, N = 917) when a sensitivity analysis was conducted excluding data from one study where there was greater than 10 year age gap between first-degree relatives and controls [26]. However it was not significant (SMD 1.19 95% confidence interval -0.99 to 3.35, N = 342) after the additional exclusion of 5 unblinded studies [24,26-29].

### Neurological soft signs in schizophrenia versus controls

This comparison comprised data on 1082 subjects from seven studies. Whilst all seven studies showed the same direction of effect, heterogeneity between studies was significant (I^2 ^93.1, p < 0.001), so the data were analysed using a random effects model. As no comparison included more than 7 studies, meta-regression was not attempted. The pooled standardized mean difference (random effects model) was 1.83 (95% confidence interval 1.28 to 2.38), indicating a large and significant effect size. This finding was stable (SMD 2.0 95% confidence interval 1.44 to 2.56, N = 955) when a sensitivity analysis was conducted excluding data from one study where there was greater than 10 year age gap between people with schizophrenia and controls [24]. It was also stable (SMD 1.60 95% confidence interval 0.64 to 2.56, N = 299) to a sensitivity analysis that, in addition, excluded 5 unblinded studies [24,26-29].

### Neurological soft signs in schizophrenia versus first degree relatives

This comparison comprised data on 1040 participants from seven studies. Whilst all seven studies showed the same direction of effect, heterogeneity between studies was significant (I^2 ^74.6, p < 0.001), so the data were analysed using a random effects model. As no comparison included more than 7 studies, meta-regression was not attempted. The pooled standardised mean difference was 0.92 (95% confidence interval 0.64 to 1.12), indicating a large and significant effect size. This finding was stable (SMD 1.07 95% confidence interval 0.79 to 1.34, N = 869) when a sensitivity analysis was conducted excluding data from 2 studies where there was greater than 10 year age gap between the group of people with schizophrenia and their first-degree relatives [24,26]. It was also stable (SMD 0.93 95% confidence interval 0.54 to 1.32, N = 389) to a sensitivity analysis that, in addition, excluded 5 unblinded studies [24,26-29].

### Publication bias

Publication bias was assessed using the Orwin fail-safe N. The Orwin fail safe N estimates the number of unpublished studies that would be required to shift the effect size towards a null result [32]. The test was used to estimate the number of missing studies with an SMD of 0 that would be required to bring the overall SMD to under 0.2, which Cohen defined as a small but significant difference. For the comparison of schizophrenia versus normal controls this would require 54. Likewise, for schizophrenia versus first degree relatives and first-degree relatives versus normal controls 31 and 23 studies would be required respectively.

## Discussion

It was hypothesized that if soft signs showed evidence of familial association, then they would be more common in people with schizophrenia versus their first-degree relatives; and in first-degree relatives versus normal controls. Both these hypotheses were confirmed. As anticipated, it was also found that soft signs were more common in people with schizophrenia than normal controls. Thus, in summary, soft signs appear to be distributed across people with schizophrenia and their first-degree relatives in a manner that is consistent with familial association.

A key limitation of this review was the finding of significant heterogeneity across all comparisons. The variance among studies could be due to factors such as variation among sample size of studies, source of normal controls, kind of first degree relatives, scales used and clinical factors such as being on medication. Insufficient studies were available to permit investigation of this heterogeneity using meta-regression. Likewise, higher scores for some signs in patients may be due to use of anti-psychotic medication and we could not conduct moderator analysis exploring the extent of its effects. Despite some similar labels, it should be kept in mind while interpreting the meta-analysis that the rating and the tasks that correspond to the individual NSS items vary between the different scales. However, all studies in the review had the same direction of effect, and the findings were stable to analysis using a random effects model. Moreover, with the exception of one comparison (first degree relatives versus controls, where the effect size remained stable but no longer statistically significant), the findings were also stable to a rigorous sensitivity analysis which ruled out those studies with poor age matching of controls, lack of reliability testing, and unblinded raters. Thus it is probable that the findings reflect true differences between the comparison groups, rather than bias or fundamental differences in study methodology. A further limitation is the possibility that the findings could be explained by publication bias. Tests for publication bias suggest that this is unlikely, but it cannot be ruled out completely.

Hence, the findings of this review add weight to the idea that neurological soft signs are an endophenotype of schizophrenia. Contrary, to other developmental markers such as 'minor physical anomalies' were early environmental factors are indicated, soft signs reflect familial association [26]. There is evidence to suggest that certain neurological soft signs correlate with region-specific structural brain deficits in people with schizophrenia [33,34]. Future research should explore the potential of these individual signs as endophenotype of schizophrenia.

Neurological soft signs can be elicited quickly, reliably and cheaply [13], they could be used in ordinary clinical settings to establish that an individual had progressed along the neuro-developmental pathway to schizophrenia. There is evidence to suggest association of neurological soft signs in relatives with schizotypal personality scores, symptom severity and neuropsychological measures [30]. The presence of higher rates of soft signs has the potential to augment the predictive power of psychopathological tests for the prodrome of schizophrenia, such as: the SIP/SOPS [35], Comprehensive Assessment of At Risk Mental States (CAARMS) [36], or Basic Symptoms [37]. Our meta-analysis highlights the meaning of neurological soft signs in the context of neurodevelopmental theory of schizophrenia. Neurological soft signs have important clinical implications and they open an avenue for future research.

## Conclusion

Neurological soft signs show a pattern of familial association in schizophrenia that is compatible with the status of an endophenotype for the disorder. The findings are based on a small number of studies. There is a need for more studies using a consensual rating tool and homogeneous sample to establish that neurological soft signs are an endophenotype of schizophrenia. Prospective diagnostic studies are required to establish how far the identification of soft signs in at risk patients can augment the predictive power of established psychopathological tests.

## Competing interests

The authors declare that they have no competing interests.

## Authors' contributions

KN and MM conceived and designed the study. KN undertook the literature search, identified potential articles, interpreted results, performed the meta-analyses, drafted and revised all versions of the manuscript. KN and DG contributed to study selection, study quality assessments and data extraction. MM contributed to study selection, study quality assessments, interpreting results, revised manuscript drafts, and supervised the study. All authors contributed to the preparation of the manuscript and read and approved the final version.

## Pre-publication history

The pre-publication history for this paper can be accessed here:

http://www.biomedcentral.com/1471-244X/11/139/prepub
